# Pursuing Public Health Benefit Within National Genomic Initiatives: Learning From Different Policies

**DOI:** 10.3389/fgene.2022.865799

**Published:** 2022-05-24

**Authors:** Suzanne M. Onstwedder, Marleen E. Jansen, Teresa Leonardo Alves, Martina C. Cornel, Tessel Rigter

**Affiliations:** ^1^ National Institute for Public Health and the Environment (RIVM), Centre for Health Protection, Bilthoven, Netherlands; ^2^ Department of Human Genetics, Section Community Genetics, Amsterdam UMC location Vrije Universiteit Amsterdam, Netherlands; ^3^ Personalized Medicine program, Amsterdam Public Health Research Institute, Amsterdam, Netherlands

**Keywords:** health policy, health plan implementation, preventative medicine, public health benefit, public health, public health genomics, precision medicine, genomics

## Abstract

**Introduction:** Population-based genomic research is expected to deliver substantial public health benefits. National genomics initiatives are widespread, with large-scale collection and research of human genomic data. To date, little is known about the actual public health benefit that is yielded from such initiatives. In this study, we explore how public health benefit is being pursued in a selection of national genomics initiatives.

**Methods:** A mixed-method study was carried out, consisting of a literature-based comparison of 11 purposively sampled national genomics initiatives (Belgium, Denmark, Estonia, Finland, Germany, Iceland, Qatar, Saudi Arabia, Taiwan, United Kingdom (UK), and United States (USA)), and five semi-structured interviews with experts (Denmark, Estonia, Finland, UK, USA). It was analyzed to what extent and how public health benefit was pursued and then operationalized in each phase of an adapted public health policy cycle: agenda setting, governance, (research) strategy towards health benefit, implementation, evaluation.

**Results:** Public health benefit within national genomics initiatives was pursued in all initiatives and also operationalized in all phases of the public health policy cycle. The inclusion of public health benefit in genomics initiatives seemed dependent on the outcomes of agenda setting, such as the aims and values, as well as design of governance, for example involved actors and funding. Some initiatives focus on a research-based strategy to contribute to public health, while others focus on research translation into healthcare, or a combination of both. Evaluation of public health benefits could be performed qualitatively, such as assessing improved public trust, and/or quantitatively, e.g. research output or number of new diagnoses. However, the created health benefit for the general public, both short- and long-term, appears to be difficult to determine.

**Conclusion:** Genomics initiatives hold the potential to deliver health promises of population-based genomics. Yet, universal tools to measure public health benefit and clarity in roles and responsibilities of collaborating stakeholders are lacking. Advancements in both aspects will help to facilitate and achieve the expected impact of genomics initiatives and enable effective research translation, implementation, and ultimately improved public health.

## 1 Introduction

Public health is defined by the World Health Organization as “the art and science of preventing disease, prolonging life and promoting health through the organized efforts of society” (World Health Organization, 2022). Following this definition, organized efforts of society that act to prevent disease, prolong life and promote health are considered as advances to ultimately benefit public health. Public health outcomes are among others shaped by a range of economic, political, behavioral, and biological factors. These biological factors entail among others the field of genomics. Genomics involves not only the knowledge of a person’s genetic makeup, but how health is influenced, both positively and negatively, by the complex interaction between genes and the environment. Over the past decades, rapid developments in the field of genomics have led to increasing application of public health genomics through its integration into healthcare and prevention (Brand, 2005; Brand, 2011; Molster et al., 2018). With the potential to significantly benefit public health, public health genomics has emerged as a topical research field and expectations from researchers, policy makers, healthcare professionals and the public are substantial (Bell, 2004; Etchegary et al., 2013; Friedman et al., 2017; Khoury et al., 2018; Molster et al., 2018; Rigter et al., 2020).

In a variety of countries, national genomics initiatives have been launched. By building on the previously gathered knowledge and practices of the field of public health genomics, many of the national genomics initiatives aim to pursue public health benefit (Belcher et al., 2020; Genomic Medicine Policy, 2022; Global Alliance for Genomics and Health, 2022). Examples of promises and aims that are stated by such initiatives include “to create the most advanced genomic healthcare system in the world, underpinned by the latest scientific advances, to deliver better health outcomes at lower cost” (Government UK, 2020) and “to improve human health through genetic research, and ultimately identify new therapeutic targets and diagnostics for treating numerous diseases” (FinnGen, 2022a) (Table 1). The former Netherlands Genomics Initiative (2003–2013) for example aimed for society and economy to benefit from the breakthroughs enabled by genomics, by concentrating talent and spawning (new) businesses (Data Archiving and Networked Services, 2022). Health was mentioned as a field to apply genomics, but at that time health benefit was not explicitly aimed for, unlike support for research and valorization. Nowadays new national genomics plans are developed in several countries often being more explicit about aiming for improved health outcomes. Summarizing, national genomics initiatives and strategies are here defined as national organized programs that aim to improve public health by (partly) using genomics knowledge and data of citizens.

**TABLE 1:** Information about population within countries and genomics initiative, and aims stated by the national genomics initiatives in literature.

Country	Population size country^a^	Initiative or Strategy	Population included in initiative (%)	Participants	Aims and goals reported by the initiative
Countries included in the literature study and semi-structured interviews:					
1.United Kingdom^b^	>67 MM	100,000 Genomes	0.14%	Patients, *via* NHS patients and their families	“Make genomics part of routine healthcare by working closely with the NHS to integrate whole genome sequencing
					Enhance genomic healthcare research by creating the largest genomic healthcare data resource in the world
					Uncover answers for participants both now and in the future through genomic-level analysis of conditions” (Genomics England, 2022)
		Genome UK	7%	Different types of patients (e.g., cancer, rare and common diseases) and healthy citizens	“Our vision is to create the most advanced genomic healthcare ecosystem in the world, where government, the NHS, research and technology communities work together to embed the latest advances in patient care
							Our goal is that patients in the UK will benefit from world-first advances in genomic healthcare through globally leading collaborations between the government, NHS and researchers, building on already successful programmes such as the 100,000 Genomes Project, delivered by NHS England and Genomics England, and UK Biobank.“ (Government UK, 2020)
2. United States^c^	>330 MM	All of Us	0.30%	Citizens	“The All of Us Research Program is a historic effort to collect and study data from one million or more people living in the United States. The goal of the program is better health for all of us.” (National Institutes of Health, 2022)
3. Denmark^d^	>5 MM	National strategy for personalized medicine—Danish National Genome Centre	1%	Patients, recruited in hospital upon suspicion of hereditary disorder	“Clear diagnosis
					Targeted treatment
					Strengthened research” (Danish Ministry of Health, 2017; Danish Ministry of Health, 2021)
4. Estonia^e^	>1.3 MM	Estonian Genome Project	15%	Citizens	“It is the aim of the Estonian Genome Project to establish a database which compiles phenotype and genotype data of a large part of the Estonian population. […] Additionally, the project will improve Estonian’s international competitiveness in high technology and have a strong educational effect on the population.” (Metspalu et al., 2004)
5. Finland^f^	>5.5 MM	FinnGen	7%	Citizens	“Project aims to improve human health through genetic research, and ultimately identify new therapeutic targets and diagnostics for treating numerous diseases.” (FinnGen, 2022b)
				Genomics to Healthcare	2%	Citizens	“Genomics to Healthcare (P6), coordinated by the Finnish Institute for Health and Welfare (THL), is a large-scale national initiative aiming to prepare the Finnish health care system for the clinical utilization of genetic risk information.” (Finnish Institute for Health and Welfare, 2022)
Countries included in the literature study only:					
6. Qatar^g^	>2.5 MM	Qatar Genome Programme	0.97%	Citizens	“Qatar Genome Program (QGP) is a national population-based research project that aims to study the genetic makeup of the Qatari population and generate large databases with the aim of introducing precision medicine and personalized healthcare.” (Qatar Genome, 2022)
7. Saudi Arabia^h^	>32 MM	Saudi Human Genome Program	0.31%	Citizens	“This program aims at reducing and preventing genetic diseases via implementing reliable screening and detection methods, and creating the physical and legislative infrastructure for development of personalized medicine. This is a substantial medical leap aimed at detecting the genes responsible for genetic diseases in the Kingdom.” (Saudi Human Genome Program, 2022)
8. Germany^i^	>83 MM	genomDE	NM	NM	“The genomDE strategy aims to give all patients access to these benefits over the long term. Along the way, ethical, regulatory and safety questions must first be clarified.” (Federal Ministry of Health, 2022)
9. Belgium^j^	>11 MM	Belgian Medical Genomic Initiative (BeMGI)	NM	NM	“The aim of the BeMGI project is to
					(i) Understand the biology of disease by exploiting the most advanced genomic tools
					(ii) Predict clinical outcome from genomic information and fulfil a pilot role towards concerted integration of genomic information in clinical care in Belgium
					(iii) Prepare the next generation of genomics researchers, informing medical practitioners, and conducting public outreach.” (Department of Economy Science & Innovation, 2022)
10. Taiwan^k^	>2 MM	G2020 Population Genomics Pilot	2%	Patients with rare diseases or cancer	“Pilot effort will sequence 10,000 genomes by end of 2020, with the goal of embedding genome sequencing in the health system by 2025.” (National Health Research Institutes Communications, 2019)
11. Iceland^l^	>365 K	deCODE	32%	Citizens	“Headquartered in Reykjavik, Iceland, deCODE is a global leader in analyzing and understanding the human genome. Using our unique expertise and population resources, deCODE has discovered key genetic risk factors for dozens of common diseases ranging from cardiovascular disease to cancer.” (deCODE genetics, 2022)

a Numbers retrieved from World Data Bank. % Calculated percentage of population aimed to include. K, thousand; MM, million; NM, not mentioned; NHS, National Health Service; NIH, National Institutes of Health. Participants were labeled as “citizens” when called “general public/population,” “individuals,” “citizens,” or when no specifics were mentioned about the included population.

b Sources United Kingdom: (Government UK, 2020; Genomics England, 2022).

c Sources United States: (National Institutes of Health, 2022).

d Sources Denmark: (Danish Ministry of Health, 2017; Danish Ministry of Health, 2021; Danish Ministry of Health, 2022).

e Sources Estonia: (Metspalu et al., 2004; Allik, 2013; Metspalu, 2015).

f Sources Finland: (SitraFund, 2015; FinnGen, 2022a; FinnGen, 2022b; FinnGen, 2022c; Finnish Institute for Health and Welfare, 2022; Ministry of Social Affairs and Health, 2022).

g Sources Qatar: (Abdul Rahim et al., 2020; Qoronfleh et al., 2020; Qatar Genome, 2022).

h Sources Saudi Arabia: (IEEE Pulse, 2015; Kaiser, 2016; Saudi Human Genome Program, 2022; ThermoFisher, 2022).

i Sources Germany: (Federal Ministry of Health, 2020; Federal Ministry of Health, 2022).

j Sources Belgium: (Department of Economy Science & Innovation, 2022).

k Sources Taiwan: (National Health Research Institutes Communications, 2019; Taiwan Human Biological Database, 2021).

l Sources Iceland: (deCODE genetics, 2022).

From a perspective of policy development, different phases can be differentiated in programs like national genomics initiatives. The public health policy cycle offers a framework to review the different aspects of start and roll out of national genomics initiatives. Phases that are distinguished in the public health policy framework are: agenda setting, policy advice, policy decision, implementation and evaluation (Jansen et al., 2021). Although in practice this order of succession is not always followed, an initiative generally starts with interest from specific stakeholders, including policy makers, which influences its place on the political agenda. Following an assessment by experts and/or decision makers, policy advice is drafted outlining if and how to proceed with a national’s genomics initiative. After a positive policy decision, the initiative embarks on implementation, for example the start of research activities, and is evaluated throughout the process and after finalizing the genomics initiative (Jansen et al., 2021).

In national genomics initiatives aiming to improve public health, the general public may be seen as a major beneficiary. Therefore, public involvement has often been regarded of high importance in shaping national genomics initiatives (Davies et al., 2017; Wagner et al., 2017; Samuel and Farsides, 2018; Holmes et al., 2019). Public involvement has shown to improve public trust and enhance the quality of the research (Brett et al., 2014; Kelty and Panofsky, 2014), as well as to ensure effective research translation and implementation (Domecq et al., 2014; Crowe et al., 2015). A recent review of public involvement in 96 national genomics programs reported public involvement (in any capacity) in only one third of them (Nunn et al., 2019). The methods (how people were involved) and tasks (what people did) of the public involvement varied considerably between initiatives and throughout the various phases. A variety of activities have been reported by Nunn et al., including but not limited to consultations, public events, formal discussions (focus groups), and surveys.

While the study of Nunn et al. (2019) found no sufficient evidence that public involvement impacted the outcome of the national genomics initiatives, Pezzullo et al. (2021) indicates that public engagement seems to lead to policy impact. More generally, according to some, it remains uncertain whether participatory and precision medicine will eventually substantially contribute to society’s healthcare interests (Juengst et al., 2012). What seems evident is that public health benefit goes beyond successful engagement and involvement of the public in a national genomics initiative.

Active genomic projects worldwide share common characteristics as well as considerable diversity in aims, scope and execution. Previous research points out that these national genomics initiatives promise to increase understanding about disease etiology, risk, prevention, diagnosis and treatment in a population in order to improve personalized (precision) treatments and prevention, as well as support genomic technological developments and data-infrastructure (Molster et al., 2018; Stark et al., 2019; Kovanda et al., 2021). These findings suggest that a variety of policies could be followed to use population-based genomics as strategy for public health improvement. While goals regarding (progress towards) health improvement are set, creating the promised health impact requires additional steps to deliver and ensure health impact. In order to guide effective and equitable implementation of genomics knowledge into health systems, governments and policy makers seem to have a unique role to play (Molster et al., 2018). Therefore, analyzing the roll out and organization of a national genomics initiative within a policy cycle may provide key information regarding implementation towards public health benefit.

Our study aims to explore to what extent and how health benefit for the general public is being pursued and operationalized by national genomics initiatives that strive to improve public health. Using a selected set of initiatives that have a stated aim of improving public health, we assess how this objective can be included in different phases of the public health policy cycle.

## 2 Methods

**FIGURE 1:**
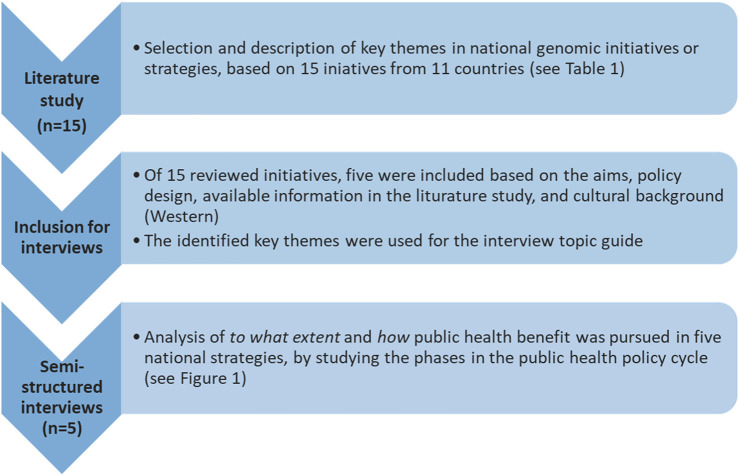
Study design. This study consisted of two phases: 1) literature study, and 2) semi-structured interviews with experts closely involved in the selected national genomics initiatives. The key themes analyzed per genomics initiative in the literature study were: aims, population, diseases, approaches/plans/actions to improve public health, stakeholders and actors, activities to ensure a successful health benefit, as well as ethical, legal, social implications (ELSI) regarding public health benefit and public trust.

Key articles were used for initial data collection (Stark et al., 2019; Kovanda et al., 2021). Available catalogues from Global Alliance for Genomics and Health (GA4GH) and Genomic Medicine Policy were consulted to identify initiatives with aims that primarily focused on health (Genomic Medicine Policy, 2022; Global Alliance for Genomics and Health, 2022). To be eligible for inclusion, the initiatives had to include an aim to positively impact the health of a population or improve healthcare. Initiatives that solely aimed to increase understanding of the contribution of genetics to disease or constructing a biobank or data-infrastructure without plans to apply that knowledge for public health improvement were excluded. Furthermore, documentation of the genomics initiatives in forms of e.g., strategy reports or information provision on websites had to be present in English to allow adequate data collation. Then, countries with national genomics initiatives were purposively sampled to represent diversity in terms of geographical location, strategies to improve public health with genomics, and different stakeholders driving the start of an initiative (e.g., government and researchers). Based on these criteria, 15 national genomics initiatives from 11 countries were selected from the available catalogues of GA4GH and Genomic Medicine Policy. For these 11 countries, a literature review was performed, followed by semi-structured interviews with experts from five purposively selected countries (Figure 1).

Data from this selection of national genomic initiatives were collected to give examples regarding the (interplay between the) different phases of the public health policy cycle and to illustrate how public health benefit could be pursued and operationalized. By pulling from the insights of the interviewed experts, the body of this work serves as exploration how the organization of a national genomics initiative can be viewed from a policy development perspective. Providing an elaborative and objective oversight on all the activities performed during a national genomics initiative goes beyond the objective of this study.

### 2.1 Data Collection and Analysis

#### 2.1.1 Literature Review

To prepare the interviews, grey and scientific literature and public domain websites were consulted to gain insight into the landscape of national genomics initiatives (Table 1). Information available in English was collected and analyzed, using the following search strings: (national genomic initiative < country name>), (national genomic strategy < country name>), (national genomic program < country name>), (<name of initiative>) or (national personalized medicine program < name country>) in Google. The searches were performed from February to August 2021. Key themes were iteratively defined and analyzed, first based on the Genome UK initiative report (Government UK, 2020) due to its broad objectives, and then supplemented with themes that were identified as key aspects upon further analysis of other genomics initiatives. The key themes analyzed per genomics initiative were: aims, population, diseases, approaches/plans/actions to improve public health, stakeholders and actors, activities to ensure a successful health benefit, as well as ethical, legal, social implications (ELSI) regarding public health benefit and public trust.

#### 2.1.2 Semi-Structured Interviews

Semi-structured interviews were conducted to gain insight in the experiences and lessons learned from experts who were closely involved in the selected genomics initiatives and have expertise in the field of genomics, healthcare, and/or policy making. A structured interview guide was developed based on the themes derived from the literature search (see Supplementary Material S1). In total, five semi-structured interviews were performed with one or two experts per interview from Denmark, Estonia, Finland, United Kingdom, and United States (Figure 1). The initiatives were selected for interviews because they reflect a variety of aims and strategies to organize a national genomics initiative and benefit public health, including improvement of patient care, embedding genomics into health services, advancement in research, and innovation in treatment. Furthermore, the organization of the initiative was taken into account to ensure that a variety of policy designs were covered in the interviews (e.g., research driven, governmentally driven). Initiatives that developed into a company were also excluded from the interviews, since a policy analysis using the public health policy cycle may not be fitting in that setting (initiative from Iceland). Furthermore, initiatives were excluded from the interviews when limited information, i.e. no public domain websites and no published reports, could be found in English (initiatives from Germany, Belgium and Taiwan). To minimize differences caused by cultural background, the authors chose to focus on initiatives from Western countries, excluding the initiatives from Qatar and Saudi Arabia.

Interviews were conducted in English. Prior to the interview, consent was collected for recording and transcribing the interview audio and archive the transcription. The recordings were deleted directly after transcription. Interviews were performed by at least two researchers, transcribed verbatim and the transcripts were checked by interviewees for accuracy.

**FIGURE 2:**
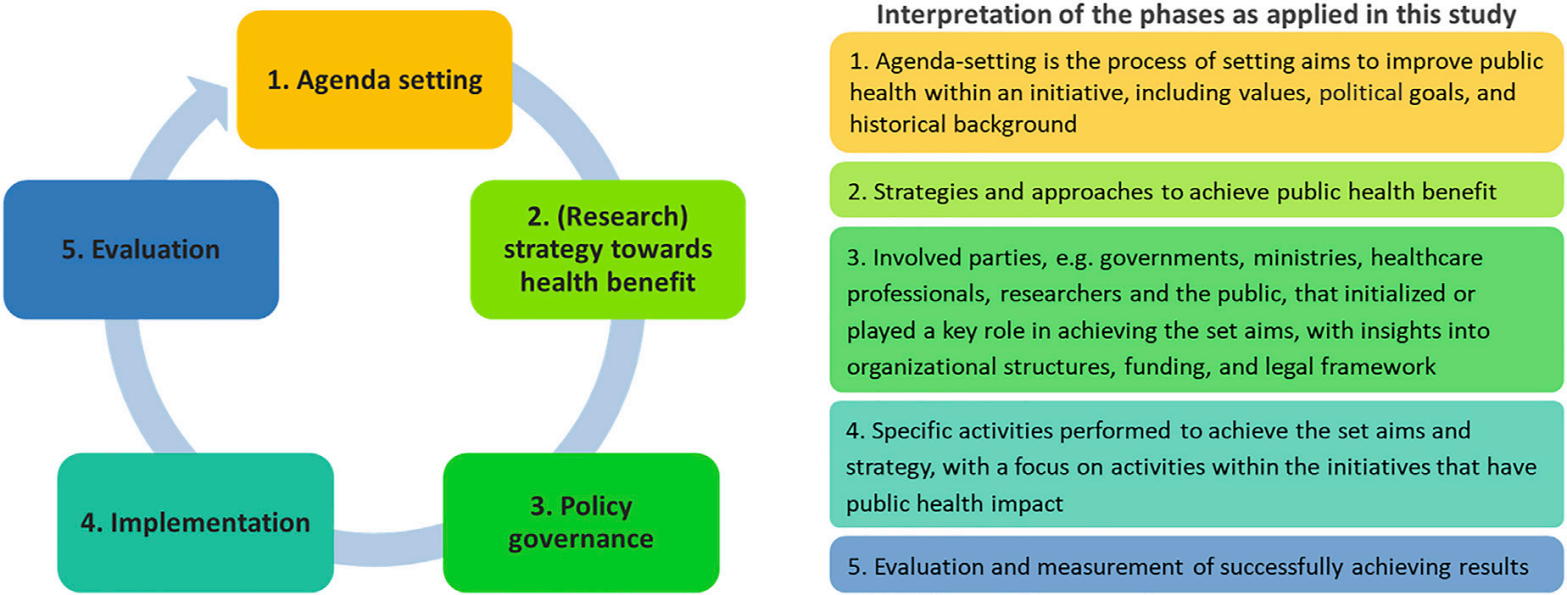
(Adapted) Public Health Policy Cycle. The public health policy cycle consists of five phases. How activities within these phases are organized, may affect the results of a national genomics initiative. Adapted from Jansen et al. (2021), Frontiers in Pediatrics.

As a theoretical framework, the public health policy cycle as described in Jansen et al. (2021) was used to extract critical aspects (Figure 2). From these, we explored to what extent and how public health benefit was being pursued in the genomic initiatives. During analysis, three researchers (SO, MJ, and TR) coded until reaching consensus on the coding tree based on the public health policy cycle. While analyzing the interview data according to the different phases of the policy cycle within the scope of this study, phase 2 “Policy advice” and phase 3 “Policy decision” appeared to be intertwined. Therefore, the original version of the public health policy cycle was adapted, with phase 2 becoming “(Research) strategy towards health benefit,” and phase 3 becoming “Policy governance.” This version was used to further analyze the results. After agreeing to the coding tree, transcripts were systematically coded by one researcher (SO). In case of doubt, researchers (SO, MJ, and TR) discussed until achieving consensus.

Specific quotes were selected (SO) if they provided relevant information about the impact on public health benefit, or discussed aspects that differed greatly from other initiatives, implying that different approaches could create public health benefit. Member checking was performed upon selection of the quotes, to check for correct interpretation and presentation of the provided information.

## 3 Results

A total of 11 countries were included, for which 15 national genomics initiatives or strategies were identified. An overview of the included countries and key characteristics of the national initiatives or strategies is given in Table 1. All these initiatives and strategies aimed, upon execution or completion, to improve the health of a population or positively impact healthcare (Table 1).

Based on the literature review, an interview guide was developed which included questions on e.g., envisioned goals within an initiative, roles and responsibilities of stakeholders, and determining whether and how an initiative will be/has been successful (Supplementary Material S1).

### 3.1 Interview Results

Interviews were performed with one policy expert and one implementation researcher from Denmark (Danish National Genome Centre), one genomics expert from Estonia (the Estonian Genome Project), one human genetics expert and one laboratory expert from Finland (FinnGen), one policy expert from the United Kingdom (100,000 Genomes), and one genomics and policy expert from the United States (All of Us).

The experts reported a variety of objectives in their national genomics initiatives (Table 2). Furthermore, they also shared insights in how the impact of a national genomics initiative could be assessed or ensured.

**TABLE 2:** Exemplary of objectives and indicators to pursue public health benefit and success in national genomics initiatives mentioned in our study.

Objectives	Indicators
• Enable excellent (large-scale) genetics research	• Scientific impact or number of publications
• Identify genetic factors that increase or decrease the risk of various diseases	• 60 000 Whole Genomes Sequenced
• Determine early onset of diseases such as cardiovascular diseases or other common complex disorders	• Analyze 5.000.000 genomes from healthy populations
• Deliver benefits to the patients	• Delivered data back to 5000 people
• Develop new treatments	• Diagnostic yield (the proportion of patients of whom you have a finding)
• Advance genomics in the healthcare sector	• A private hospital that provides risk assessment on cancer
• Maintain public trust and confidence	• Building a complete infrastructure
• Kickstart the genomics industry	• Building a genome centre

If and how public health benefit is being pursued within national genomics initiatives is the result of interplay in activities throughout the different phases of the public health policy cycle (Figure 2). A variety of ways to operationalize public health benefit within national genomics initiatives were found in all phases of the public health policy cycle: agenda setting, (research) strategy towards health benefit, policy governance, implementation, and evaluation.

#### 3.1.1 Agenda Setting

The insights that the experts provided indicate that agenda setting of national genomics initiatives was influenced by the presence of strong political will or drive and demands from other stakeholders, as well as the country’s history and existing societal values.

Several goals and interests of key initiators and stakeholders were identified as incentives to start a national genomics initiative. These goals fitted in the expectations that genomics can create public health benefit. Improvement in public health was reported as goal itself, combined with goals that ultimately steer towards public health benefit through organized efforts in healthcare and research:

“The Estonian initiative was just to make a large biobank in order to be competitive. Competitive in research, and also use data in improving public health.” Estonia

“We for sure think that the patients are the most important stakeholder […]. If this initiative doesn’t benefit the patients, and if it doesn’t gain legitimacy from the patients, then it’s not really worth it.” Denmark

“This is an initiative that was initiated by researchers […]. There are two main goals. The first one was to be able to produce a large enough dataset that enables excellent genetics research. And then the other goal is, of course, to utilize the data to be able to identify genetic factors that increase or decrease the risk of various diseases.” Finland

Drive or demands from different stakeholders including society, key politicians, and researchers also reported the initiation of a national genomics initiative:

“Society wants to get better medical care, and this is why we are providing the scientific base and helping medical institutions, because the university is not providing medical care itself as they just do science and teaching.” Estonia

“One person who really wanted this to happen was President Obama […] he proposed it in a major announcement, and then the Congress got behind it, we got the money, and off we went.” United States

In addition to the drive and demands from different stakeholders, it was often mentioned that important societal values within a country were intertwined with the agenda of a genomics initiative. These values include, for example, equity in research and health care, public trust, or transparency in research:

“There are so many threads in here that are societal, that are about equity and some issues that are bigger than science in many ways, […] so a lot of effort has always gone in ‘All of Us’ to think about, to study, to anticipate societal concerns around privacy, security, discrimination, and so forth.” United States

Experts discussed that the history or tradition of a country could be an important factor to address societal values, and could therefore influence decisions made within a national genomics initiative:

“Finland has this tradition of people who are very interested in research and very supportive. […] Starting this kind of initiative means that we do not want to lose the trust, so that is also one of the main reasons why we are wanting to do this as transparently as possible.” Finland

As stated by these experts, maintaining public trust requires additional efforts regarding transparency in research activities towards the population.

#### 3.1.2 (Research) Strategy Towards Public Health Benefit

Although similarities in (sub)goals that lead to public health benefit could be found, the strategies to achieve these were different. Some initiatives had a rather research-based strategy to generate data, information, and knowledge to increase the understanding of population health and disease etiology.

Another strategy mentioned by the experts was a more translational strategy, focusing on bringing new or existing knowledge and developments into practice, for example by developing a citizens’ support system that produces a personal health report. Within the translational strategies, the following subgoals were reported: embedding genomics in healthcare, prevention of disease, improved diagnosis, improved or personalized treatment, and development of innovative treatments or technologies. Yet, both strategies fit with the idea that national genomics initiatives benefit public health through the art and science of preventing disease, prolonging life and promoting health, as “public health” is defined by the WHO.

“Our focus is on the patients, so the most important thing is to help the patients and to make sure that the patient gets the correct treatment.” Denmark

“We have the common goal of being able to help people and for this we need the pharma industry. We need new treatments, so we hope that the project will lead to new treatments.” Finland

“The latest strategy, […] is what we call the Infinity loop. On the left-hand side, the kind of health care service works, and we support them. The data then goes to the Genomics England side and then we provide researchers access to it as secure environment. Then the findings very quickly go back into the health service in this kind of Infinity loop.” United Kingdom

The Infinity loop strategy, as discussed by the experts from the United Kingdom, illustrates that advancements in genomics research and bringing these advancements into practice is an intertwined process, which requires a collaboration between research and health systems.

Some national genomics initiatives or strategies target specific areas for impact, for example, diseases that are endemic or prevalent to their country:

“The main research focus is genetic risk factors that actually are only present in the Finnish population and that cannot be identified anywhere else because of this bottleneck population effect.” Finland

The focus of this initiative illustrates an interest to improve health of the national population specifically. In comparison, a focus on specific diseases and patient groups, both in research and in implementation into health systems, was also found, including cancer, cardiovascular diseases, pharmacogenomics, and rare diseases:

“In 2023, we hope to have the first services for cardiovascular disease, cancer, and pharmacogenomics for the primary care providers.” Estonia

“The four aims of the project were to deliver 100,000 genomes from NHS patients, to identify the causes of rare diseases [and] cancer, and to provide opportunities for research and industry. […] each one of them was equally important. So, to deliver benefits to patients, to provide opportunities for research, to maintain public trust and confidence, and to what we call kickstart the genomics industry.” United Kingdom

Strategies to improve public health were often approached through joined forces between multiple fields within society, e.g., research, industry, public, and healthcare. Combining all these fields and formulating corresponding goals seem an important aspect within strategies to yield success.

#### 3.1.3 Policy Governance

Different aspects that influence governance within national genomics initiatives were found to be critical in this phase. Here, we focus on drivers and funding of an initiative, legal frameworks, and the roles and responsibilities of involved stakeholders. While these aspects may not all seem to be directly linked to public health benefit, they provide insight in how the organized efforts are expected to affect the initiatives that ultimately aim to improve public health.

Firstly, drivers and ownership of the initiatives differed across our study set. Some initiatives were fully owned by the government, while others were identified as academic or public-private initiatives:

“The National Danish Genome Centre is an agency in the ministry of Health.” Denmark

“We were very happy being an independent institution, who is just outside of faculties. Just under the Rector of the University. But since 2019, the Estonian Genome Center is part of the Faculty of Science and Technology of the University of Tartu.” Estonia

Funding for the national genomics initiatives came from a variety of sources, including private funding, governmental financing, or funding from outside of the country. The funding source did not always affect the organizational structure. For example, the government-owned initiative in Denmark receives an annual national budget as part of a political agreement. Yet, the majority of funding was from a donation by the Novo Nordisk foundation (Novo Nordisk, 2022) (which has no decision-making role in the initiative):

“It [the National Danish Genome Centre] is funded mainly by a private fund called Novo Nordisk foundation. This is a very big fund in Denmark, funding health research, and they have given us around 130 million euros. […] That’s extremely unusual in Denmark.” Denmark

“In the first step, we actually raised private money from the US and used very little government money at all […] Since 2007, the Estonian government is the principal funder of the Estonian Genome Center. In the last five years, most of the money for the biobank is coming from the Ministry of Social Affairs. Of course, we have to apply and win research grants and attract private funding in addition to the government funds.” Estonia

The legal framework of the country seems to largely influence the governance of its national genomics initiative. Often, regulations were reported to impact the organization of the initiative, including roles and responsibilities of the stakeholders regarding specific tasks, e.g. data-management and access, data protection, or recruitment of participants:

“It was a political decision to start the initiative, that was made a political agreement. Following the agreement, they made amendments to the health law which made the construction of the national genome center possible. The political decision was based on input from researchers, clinicians, their citizens, etc.” Denmark

“In 2008, the US passed a law called the ‘Genetic Information Non-Discrimination Act.’ […]When it comes to employment and health insurance, you cannot be discriminated against based on genomic information.” United States

Interviewees from both Finland and Estonia stated that the existing legal framework warrants that (research into) public health benefit was ensured within the national genomics initiative:

“The law that tells us we have to protect the data, to analyze the data and perform research, and to use the data to improve the public health. These are three things described in the law and this is why the biobank was basically created.” Estonia

“There are a lot of research regulations that are important for us, but the Biobank Act is the most important one. […] the Biobank Act enabled broad consent. Before that, we always had to ask for consent for a specific research project, for example breast cancer research. Now the broad consent is just that the participant consents that their sample data can be used for any future medical research project that is approved by the biobank.” Finland

A variety of stakeholders were identified that held leading roles and responsibilities within national genomics initiatives. This implies that different stakeholders within society were involved to translate advancements of genomics towards public health benefit. The most frequently mentioned stakeholders were governments and politicians, national health services, researchers, biobanks, genome centers (sometimes specifically built as part of the initiative), clinicians, patients, the public, and industry. Although all parties seemed to hold important tasks, interviewees often emphasized the involvement of the government and the public as essential for the initiation and success of national genomics initiatives:

“I guess you have to win over the government first. Otherwise, because it’s so much money and the government are not supporting, there is no way to do it. […] But the most important thing is you have to get people over, because finally people have to come and donate blood. The information they get is only the promise that in the future it gets better.” Estonia

Remaining transparent in research and ensuring that the public participates in the initiative were mentioned as arguments to involve the general public and patients in any form. Another argument to involve the public was to ensure that the aims and activities of an initiative are in line with the public’s wishes and expectations. In some settings, the patients could influence which disease groups should be looked into with the national genomics initiative:

“We decided to include patient-citizens and obviously clinicians in deciding which groups we should look into. Therefore, we had a round where people could report to clinicians, as well as citizens who could report which groups we should look into […].” Denmark

This indicates that the general public and patients may influence research translation, including how public health benefit is yielded and which policy decisions are made. To do this, the interviewed experts stated that patients and citizens fulfilled different roles affecting governance and structure within a national genomics initiative, including participation in advisory and agenda-setting committees:

“The participants panel now has a key role in the governance of several of the big decision-making committees.” United Kingdom

The perspective of participants was described as important and refreshing, since they e.g., challenged experts to rethink about common practice, and required experts to explain the choices they made within the initiatives.

#### 3.1.4 Implementation

A variety of plans and activities to pursue public health benefit in the implementation phase were reported in all the national genomics initiatives. The operationalization of public health benefit was found to be in different stages in national genomics initiatives, as some experts discussed that the first steps towards e.g., implementation of genomics in research setting have been made, while other experts reported that these steps are still in preparation.

The diversity within the implementation phase will be illustrated below, through presentation of different activities discussed by the experts. For example, the expectations of genomics to benefit public health were translated into activities to return genetic results to participants:

“We are running WGS now and we are actually reporting back to the patients already. Now we have five regions in Denmark. And we are reporting back to patients in two regions. The last three regions are close to getting all that data processing agreements in place.” Denmark

Yet, the insights that experts provided indicate that reporting back genetic results comes with additional efforts. To maximize potential health benefits of genomic research in a comprehensive and equitable manner, recruitment of people from diverse races and ethnicities was highlighted as key:

“It is time to have data and research that reflects the diversity of the United States population, and so […] 70%–80% of the people who have been enrolled in ‘All of Us’ so far are from groups that have been traditionally underrepresented in biomedical research. […]. A lot of them are ancestry related, […] we wanted to capture people with different social economic backgrounds, rural versus urban, […]. With ‘All of Us,’ the value is to get genomic data from ancestral groups that we do not currently have. […] So, in order to really strengthen our ability to implement genomic medicine in a comprehensive way, we first need genomic data from individuals from different ancestries.” United States

Additional approaches were expressed as required to understand disease etiology and health needs in underrepresented groups. Yet, the efforts to include them were faced with additional barriers:

“I think the issue that we’re still grappling with is how to get to hard-to-reach groups […]. [For example] we know roughly what our census tells us about the diversity of our population, we are not so clear about what their health needs are. So, it may be one thing to have say, you know, 5% of people who are from […] minority populations, but what if they have higher [or lower] health care burden in cancer, or particularly in rare diseases because of consanguinity? So, we're always keeping a very close eye on that.” United Kingdom

In addition to barriers regarding recruitment of (specific groups of) participants, experts from the United Kingdom reflected on challenges to communicate results to participants:

“Something like 80% of people would like that feedback. We haven’t done it yet. It’s just too complicated. Every time we think we’ve done the bioinformatics, a new disease association or new data comes up so that needs to be fixed, and then we have to understand how we would do that clinically for the 1% to 2% of people who are having a finding? […]. So, lots of communications issues in the clinical issue.” United Kingdom

Furthermore, the expert from the Estonian initiative stated to have grappled with how the results should be communicated to match this with the expectations of the participant:

“Feedback is important. I was surprised that some people were not happy […]. So, I was asking what was the problem? ‘Nothing, you know they didn’t tell me anything,’ So, I said ‘Look, that is good news, you do not have a high risk of breast cancer, or cardiovascular disease, no Parkinson, no nothing. So, you should be really happy, not just worried that you had nothing, it’s good news’. And then these people started to think ‘you are right.’ They said, ‘I’m really happy that I had no news from this thing because any news would be bad. But, in general, over 95% of people were very positive about the feedback they received and didn’t regret it even 6 month later.” Estonia

These insights imply that there may be tension between the expectations and true impact that can be delivered through advancement in genomic research.

In addition to returning results to participants, other efforts to improve health were reported, including translating new insights into research or healthcare, broader application of Whole Genome Sequencing (WGS) in healthcare, and implementation of polygenetic risks scores.

Many experts described the development of a data-infrastructure as key to enable genomics use within health systems:

“Personalized medicine is often very data driven and data heavy, so that needed some change of the infrastructures and organization in the healthcare system […] and so we needed somehow to be able to collect and store genomic data. That was like the first big task, and that is still the main task […].” Denmark

In order to embed genomics into healthcare, multiple interviewees stated that specific attention should be paid to involving and educating the medical community:

“So, you also have to engage the health care professionals. This is not actually just the doctors, it has to include the nurses, the pharmacists, everyone.” United States

Additionally, the Danish National Genome Center highlighted that to ensure successful implementation of personalized medicine in the healthcare system, it is important to proactively secure the right expertise and workforce to perform the interpretation of WGS and other comprehensive genetic tests. For this, the development of standards for the interpretation of results and criteria for stratification of patients was necessary.

#### 3.1.5 Evaluation

Depending on the aims and strategies, different methods to evaluate achievement of envisioned goals and success towards public health benefit were reported. A variety of elements were identified within the evaluation process that provide insight into how goals are strived to be achieved, including setting general milestones and deadlines, determining deliverables before and during execution of the initiative, and setting requirements to receive funding:

“We have deliverables and milestones set in our consortium agreement and deadlines […]. From the beginning, we have had a project start and an end date for the initiative […] we have set structure for the project and set goals.” Finland

The number of genomes collected was often mentioned as indicator of progress for national genomics initiative:

“As part of the agreement with the [donation from the] Novo Nordisk foundation, we have to make 60,000 WGS by the end of 2024. You could say that’s kind of the quantitative measure we have.” Denmark

Additional information was collected to monitor the representation of the collected genomes, including e.g. geographic background or patient groups:

“We used to have many complicated dashboards here, we aimed for 100,000 whole genomes […] We kept a close eye on whether we had underrepresentation geographically as well as in the population mix for a long time” United Kingdom

Keeping a close on these additional characteristics implies that equity in research, a value addressed in “agenda-setting,” was monitored during data collection of a national genomics initiative. Provision of samples and consent may also be an indicator for public trust, indicating that this value could also be monitored during the roll out of a national genomics initiative:

“And that is also one important way to measure the trust, because we are assuming that if we lose the trust, people stop providing their samples, providing their consents, and it has been very stable throughout the project.” Finland

As stated by the expert from the United Kingdom, achieving the aimed number of genomes was not seen as sufficient to determine success in their national genomics initiative:

“You can’t just hit the target and miss the point. You could go and get some genomes from anywhere. But if you don’t have it embedded properly with the data and the data aren’t high quality, or you don’t have consent, then you missed it. You missed the point. This is not just a numbers game.” United Kingdom

As can be seen in the reported goals, many genomics initiatives aim to improve public health, by either preventing disease, prolonging life and promoting health. Yet, the difficulty in currently assessing the public health impact was also mentioned:

“The third criteria, which obviously takes decades to measure is: Are you making scientific discoveries that are improving human health? Are you making discoveries that are changing clinical practice? Are you making discoveries that you could point to and say that this is improving people’s lives? […] Science is not a sprint; it is a marathon. To really be able to measure impact on public health, you have to be willing to wait several decades.” United States

While it may be too soon to determine to what extent and how public health benefit is created within a national genomics initiative, intermediate goals and indicators are often reported by interviewees. Indicators to evaluate research and technical progress include building an infrastructure that enables clinicians and researchers to use genomic technologies and data, collaborating with industry partners, and publishing novel scientific discoveries:

“The earliest success will come from whether people are using the data. That is the earliest success. If you build something and nobody uses it, well, then you know you’re failing.” United States

Furthermore, indicators to evaluate progress towards public health impact were also reported, such as diagnostic yield, reporting genetic results to patients, making discoveries that change clinical practice, or developing new treatments:

“Another metric is our diagnostic yield, as we call it, the proportion of patients where you have a finding. […] And the other success metric, we’re giving ourselves a hard task, is that we had an optional consent in the 100,000 Genome project for people who wanted to know additional findings.” United Kingdom

“The very important measure, and also obviously as secondary use, you could say that researchers gain access to our data and then they can actually use this to develop new treatments for patients.” Denmark

The latter statement of the Danish experts indicates that, in order to ultimately prolong life, promote health and benefit public health, e.g., through developments of new treatments, efforts in research are necessary to make those improvements possible.

## 4 Discussion

Public health genomics involves the translation of genome-based knowledge and technologies into public health (Bell, 2004; Friedman et al., 2017; Khoury et al., 2018; Molster et al., 2018; Rigter et al., 2020). This emerging field has heightened expectations for the advancement of personalized and precision medicine among researchers, healthcare professionals, policy makers and the public. In this study, we explored how public health benefit is being pursued in selected national genomics initiatives, using an adapted version of the public policy health cycle.

This study showed that the initiation and implementation of current national genomics initiatives are shaped by an interplay of aims, cultural values, history and push from various stakeholder groups. Further setup and organization of initiatives was found to depend on the governance structure as well as the chosen strategy to achieve public health impact. In general, strategies from the national genomic initiatives that we studied here are varied—ranging from more research-based strategies to translation-based strategies, or a combination of both—with a general focus on specific diseases or application areas.

In this study we found little evidence of true operationalization of public health benefit across the various public health policy cycle phases in national genomics initiatives. Therefore, as phrased by Juengst et al. (2012), there is risk that the widespread and compelling appeal of personalized genomic medicine’s vision and potential virtues ultimately do not contribute to society’s health care interests. Although the general aims and strategies to achieve public health impact are formulated in most national genomic initiatives, the research translation and implementation seems to be not always clearly outlined in the different aspects of the public health policy cycle.

In addition to improved public health, one of the aims or incentives that was often referenced by the interviewees was to stay ahead of competition. Yet, it was not always made clear why that is considered important. Underlying ambitions and arguments to start genomics initiatives and improve public health, such as for-profit development of technologies or treatments, may not be brought to light completely in this study. It would be interesting to study how this incentive may influence the organization and policy decisions made within a national genomics initiative, and whether and how this incentive affects the operationalization of public health benefit.

Moreover, the evaluation of actual public health benefit seems to lack well-defined indicators. Many experts stated that the amount of genomic data collected can be used to measure quantitative progress. Yet, as stated by one of the interviewed experts, “you can’t just hit the target and miss the point,” suggesting that the success of a national genomics initiatives aiming towards public health benefit should not only be measured by a set amount of genomic data. Experts in this study pointed to other indicators to assess progress and effective roll out of a national genomics initiative, including data-infrastructure that enables clinicians to improve treatment, diagnostic yield, or the development of novel treatments.

Generally, research-based strategies are not primarily pursuing direct impact on public health, yet their strategy may be seen as efforts to prepare the delivery of public health benefit. Translational strategies varied, and were more directed at delivering (meaningful) results to patients and citizens. Implementation of strategies is often accompanied by public involvement and recruitment, designing and building data-infrastructure, as well as several strategy-tailored activities, including education of healthcare professionals and establishment of a (national) biobank. Approaches and activities to pursue public health benefit differed. In some initiatives, for example, patients were involved in deciding which diseases should receive priority attention from the genomic programs, while in other initiatives this decision making role was set aside for experts.

As shown by the challenges faced by e.g., Estonia and the UK regarding reporting results back to participants, it seems pertinent to pay attention to how public health benefit is operationalized and what additional activities and corresponding policy decisions are necessary to ensure this. Examples include, but are not limited to, effective communication with the patients, educational support for healthcare professionals, clarity about the meaning of complex genetic test results, and guidelines about follow-up treatment.

Generally, advancements in science that are translated into healthcare should be accompanied by careful ethical and social evaluation. National genomic initiatives are no exceptions, and also require clarity in aims and transparency in research. Dialogues involving all stakeholder during the various phases of the policy cycle can also promote responsible implementation and public trust.

Public trust in science, which was expressed by many experts as an important goal in their initiative, seems to demand transparency. Therefore, the aims of national genomics initiatives should be clear from the beginning or, in case of change due to advancements, adapted in a transparent way. The achievement of these aims are in this study shown to be evaluated as follows: scientific insights are assessed as publications and patents; infrastructures for data storage and future research assessed as infrastructural capacity achieved; and public health benefit assessed as new diagnoses for unsolved genetic diseases, pre-symptomatic diagnoses made allowing for early interventions, and health gain through timely prevention or risk management. Because many initiatives are still ongoing, the full impact of genomics on public health may not be realized for decades. The development of tools and methodologies to realize and determine effects are still evolving. Yet, we argue it is not too early to evaluate the effectiveness of activities meant to measure the progress in public health benefit.

The policy cycle framework is designed as a learning system, to enable adjusting policy to relevant developments. By feeding back outcomes of evaluation to the initial phases of the cycle, strategies are ensured to maintain relevancy. To achieve a true feedback-loop in the policy cycle of national genomic initiatives, initiators should not only set clearly-defined goals, but also pre-determined milestones and indicators that can be used to measure the progress of the chosen strategy towards health improvement. As stated by the interviewed expert from the United States, long-term effects and results of initiating and executing a national genomics initiative, including public health impact, seem difficult to determine at this early stage. To ensure that public health benefit can be measured effectively, both short and long term, it is important that pre-determined (sub)goals with accompanying requirements are set. This should include how goals are aimed to be implemented, and what data needs to be gathered in order to determine whether a national genomics initiative has been successful in improving public health.

Beskow et al. (2001) proposed a blueprint to integrate genomics into public health, consisting of research inquiries that require attention*.* Applying this blueprint may provide a way to effectively integrate genomics into public health throughout the different phases of the public health policy cycle that can be found in a national genomics initiative. Khoury et al. 2018 also called for specific attention regarding system management, acknowledging that public health infrastructure has a vital role as both support for and a conduit between research and practice. This call seems to be partly met by the majority of the included national genomics initiatives, as many experts expressed the importance of a data-infrastructure for the collection, analysis, and reporting of genomic results. Additionally, the UK’s Infinity loop-strategy demonstrates a seemingly ideal interplay and data flow between health care services and researchers, promoting simultaneous utilization of genomics. In strategies like these, which other experts also referred to as a learning health care system (Wouters et al., 2020), system management could play an essential role in integrating genomics into public health practice, when accompanied by ongoing evaluation and subsequent refinement of the requirements and policies that ensure beneficial impact and responsible implementation.

General benefits and risks of (aspects of) national genomics initiatives can perhaps be distilled from similar implementation processes. For example, experience gained from implementing clinical decision support systems could be translated to setting up a data-infrastructure embedding genomics into health care (Sutton et al., 2020). Proposed efforts by Sutton et al. to ensure benefits overcome potential risks of setting up these infrastructures include prioritizing evidence-based genomics-disease interactions and adequate training for users of the support system (e.g., health care providers).

In a recent commentary, the WHO and member states acknowledge that to accelerate and amplify impact on population health, utilization of digital interventions, tools and systems to deliver clinical, public health, and data recommendations offer potential (Mehl et al., 2021). However, it was discussed that interoperability, continuity of care, optimized data use and accountability in health data systems is hindered due to limited translation, operationalization, and incorporation of health and data recommendations and lack of guidance on both technology and content level. Their proposed guidelines may serve as a basis for an effective approach for national genomics initiatives towards systematic, transparent, and testable data-infrastructure development with digital systems at the country level.

As stated by multiple experts, involvement and support of both the public and the government are crucial to a successful start and execution of a national genomics initiative. However, based on this study and the study of Nunn et al. 2019, it is not clear how involvement of the public impacts the envisioned goals of a national genomics initiative beyond retaining public trust. Different approaches to inform and involve the public exist. Avard et al. (2008) have distinguished indirect and direct public involvement activities. They described indirect public involvement as a one-way communication, such as surveys or consultations. Direct public involvement was described as a two-way communication process, with activities including citizen workshops, dialogues, and deliberative and consensus conferences. These approaches and activities may prove suitable for different objectives, e.g., informing about vs. co-designing research. Additionally, management of public expectations is important to avoid erosion of public trust, due to uncertainties in the delivery time and form of potential health benefits (e.g., improved diagnosis of hereditary disorders or personalized medical treatments).

Furthermore, other stakeholder groups may hold crucial roles in a successful roll out of a national genomics initiative, including but not limited to health care providers, pharmacists, or policy makers. In order to deliver the promised goals regarding public health benefit, policy makers and governments have a unique role to play (Molster et al., 2018). The complex interplay between multiple stakeholder groups with their own roles and responsibilities should be acknowledged and receive further attention. Complex structures of multi-organizational collaborative approaches can be found in national genomics initiatives. Gil-Garcia et al. (2019) called for clarity in roles and responsibilities in government inter-organizational collaboration and information sharing initiatives, and conclude that this is a critical factor for success. In light of the current study, the roles and responsibilities of stakeholders should be assessed and clarified for each of the different phases of the public health policy cycle and corresponding milestones or indicators. In this, specific attention should be paid to the parties responsible for evaluating the impact of a national genomics initiative on the envisioned goals and public health impact in the long run.

## 5 Limitations

This study faces several limitations. The literature review was restricted to information about national genomics initiatives available in the English language. Therefore, some national genomics initiatives may have been overlooked, e.g., due to absence or difficult to find information, while others may have been partially reviewed. Yet, the main findings within this study were collected during the interviews. The national genomics initiatives that were subject of the interviews reflect the diverse landscape of national genomics initiatives. Therefore, we expect that combining the explorative literature review and interviews from different perspectives has sufficiently enabled us to illustrate possible operationalization of public health benefit in national genomics initiatives.

The information obtained by the authors was gathered during interviews with experts who are involved in their countries’ initiative, likely resulting in a limited and perhaps biased view on all aspects. As the execution of a national genomics initiative requires collaboration from multiple stakeholder groups, it would have been insightful to also have included other experts representing different stakeholder groups per country. In doing so, we could have included varying perspectives about the pursuit and operationalization of public health benefit within the different phases of the public policy health cycle, and which indicators were evaluated. However, the interviewed experts were all closely involved in their countries’ national genomics initiatives, and were therefore able to provide important insights in the different phases of the public policy health cycle.

## 6 Conclusion

National genomics initiatives hold the potential to benefit to public health. This study showcases several different policies that currently pursue public health benefit through national genomic initiatives. Sometimes, public health benefit is directly pursued within national genomics initiatives, with goals set to improve prevention, diagnosis, and interventions, while in other initiatives, public health benefit is seen as a future goal of current research activities that are aimed at generating data and knowledge. To date, the development of international and standardized tools, methods, and data sharing is necessary to operationalize the anticipated beneficial impact of genomics initiatives on public health. Furthermore, evaluation of actual public health benefit can benefit from well-defined indicators, also to compare between countries and draw on lessons learned. In order to achieve the envisioned goals of national genomics initiatives, the indicators should not only be operationalized, but it should also be clear who has what role and responsibility throughout the different phases of the public health policy cycle, especially regarding evaluation of the public health benefit within a national genomics initiative.

## Data Availability

The datasets presented in this article are not readily available because participants only gave consent to publication of the data included in the article. Raw data cannot be anonymized because statements can be traceable, to specific experts/expertise. Requests to access the datasets should be directed to suzanne.onstwedder@rivm.nl.
