# Supplementary material for: Pursuing Public Health Benefit Within National Genomic Initiatives: Learning From Different Policies
Source: Front Genet. 2022 May 24;13:865799. doi: 10.3389/fgene.2022.865799 (PMC9171010; doi:10.3389/fgene.2022.865799)
Supplement: Supplementary file 1 [file DataSheet1.docx]

Supplementary Material 1: interview guide interviews

**Interview questions:**

*Culture: the way of thinking*

1. What is the goal of your national initiative?
   1. Why was this goal chosen?
   2. Why is collecting (and sharing) genetic data important to achieve this goal?
2. How does this initiative complement health policy and public healthcare ambitions in your country?
3. How do you make sure that your countries’ diverse population is represented, as well as possible, in the datasets?
4. Did you include society perspective in the developing process of the initiative?

*Structure: the way of organizing*

1. Looking to the aim of the initiative, by whom is your countries' national initiative initiated? (*Focus: roles and responsibilities*)
2. What kind of regulations were followed for initiating and continuing the national initiative?
3. What are, according to you, the most important stakeholders/ actors?
4. To initiate
5. To execute
6. Were other local genomic initiatives (governmental or private) involved while developing this national initiative? (*Think of pre-existing biobanks or genomic consortiums.*)
7. What is the role of the government?
8. How can government contribute to the success of the genomics initiative?
9. Which ministries?
10. What is the role of public health organizations?
11. How do you determine whether the initiative was successful?
    1. What was/is seen as a success?
    2. How do you measure success? And when?
    3. How do you determine the deadline for your initiative?
12. Does this [name initiative] collaborate with other countries?
    1. If so, which agreements are involved in this collaboration?
    2. And what is important to consider, once there is a collaboration?
13. Where does the funding from the initiative come from and how is it invested during the period of the initiative? (Sources of investment and budget allocation)
14. How did the organization/structure affect the outcomes/achievement of the aim?

*Practice: the way of doing things*

1. For starting the initiative, which key elements are important for starting an initiative?
2. What went right in the initiative, that you would also advise us and looking back, what could have been changed in the initiative?
3. What challenges were faced during the initial phase of the initiative?
4. What are important actions/activities that helped?
   - 1. To initiate
     2. To achieve the aim or successful rollout of the initiative?
5. How did you include society perspective/the public in the developing process of the initiative?
6. How does [name] initiative create and ensures public trust regarding sensitive (genomic) data security and privacy?
7. Which steps were taken to ensure data security? By whom?
8. Who has access to the data?
9. What happens with the public's data once an initiative programme is finished?
10. What are the future plans of the initiative?

*Final*

1. What are three important considerations to successfully start or complete a national genomics initiative?
